# Large-scale reverse docking profiles and their applications

**DOI:** 10.1186/1471-2105-13-S17-S6

**Published:** 2012-12-07

**Authors:** Minho Lee, Dongsup Kim

**Affiliations:** 1 Department of Bio and Brain Engineering, Korea Advanced Institute of Science and Technology, 291 Daehak-ro, Yuseong-gu, Daejeon, 305-701, Korea

## Abstract

**Background:**

Reverse docking approaches have been explored in previous studies on drug discovery to overcome some problems in traditional virtual screening. However, current reverse docking approaches are problematic in that the target spaces of those studies were rather small, and their applications were limited to identifying new drug targets. In this study, we expanded the scope of target space to a set of all protein structures currently available and developed several new applications of reverse docking method.

**Results:**

We generated 2D Matrix of docking scores among all the possible protein structures in yeast and human and 35 famous drugs. By clustering the docking profile data and then comparing them with fingerprint-based clustering of drugs, we first showed that our data contained accurate information on their chemical properties. Next, we showed that our method could be used to predict the druggability of target proteins. We also showed that a combination of sequence similarity and docking profile similarity could predict the enzyme EC numbers more accurately than sequence similarity alone. In two case studies, 5-flurouracil and cycloheximide, we showed that our method can successfully find identifying target proteins.

**Conclusions:**

By using a large number of protein structures, we improved the sensitivity of reverse docking and showed that using as many protein structure as possible was important in finding real binding targets.

## Background

Identifying disease genes and target proteins of drugs is a critical step in drug discovery. Once the disease genes are identified, designing lead compounds which can modulate those genes or the protein products may lead to a successful new drug. The growth of the number of available 3D structures of proteins and computing power has enabled high-throughput computational screening of lead compounds, which is known as virtual screening. Conventionally, these virtual screening methods have focused on searching chemical space for chemicals that can specifically bind to a protein target [1].

Complication in this structure-based drug discovery strategy is that there may exist unknown off-target proteins that can bind to the lead compounds unexpectedly, which undoubtedly poses some difficulty such as severe side effect, but also provides a new opportunity. Upon discovering novel drug targets for existing drugs, we can expand indications of the drugs by drug repositioning. Motivated by this, reverse (or inverse) docking approaches have received increasing interest to find unknown targets of natural products and existing old drugs [2-4]. In reverse docking, one tries to find the protein targets which can bind to a particular ligand.

In previous researches, based on an assumption that the number of predicted potential protein targets [5] is quite low compared to the number of genes, they tried to find new drug targets among a relatively small number of potential target proteins. For example, a reverse docking study by Gao et al. used ~1,100 targets [6], and that by Hui-fang et al., used 1,714 targets and 8 compounds [7]. However, this may cause poor coverage of the protein structure space in reverse docking. Moreover, their only intended application of their reverse docking methods is to find the targets of drugs. On the other hand, various approaches including statistical method using sequence and structure similarity [8], calculating binding site similarity [9,10], and prediction of druggability by descriptors [11] have been developed.

Here, we present a large-scale reverse docking study. The main difference from previous studies is that we used all available protein structures in human and yeast. To our best knowledge, our docking profile contains the largest number of protein structures. The reverse docking profile was merged into a matrix which can be easily interpretable. We showed the some properties of the large-scale docking profile and demonstrated usefulness of these docking profile data. We also developed several new applications such as predicting druggability of protein targets and protein function prediction based on docking profile similarity. We discussed two interesting case studies, 5-flurouracil and cycloheximide. Especially, we successfully demonstrated that using as many protein structures as possible was important in improving the sensitivity of reverse docking and finding real binding targets.

## Results and discussion

### Data structure

**Figure 1:**
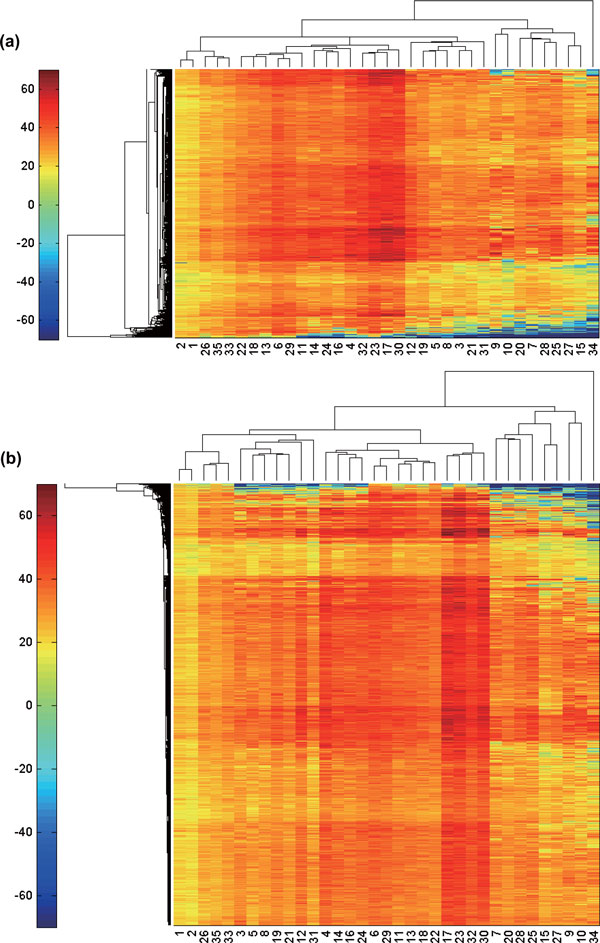
**Color mapped docking fitness profile matrix in (a) yeast and (b) human**. The elements represent corresponding docking fitness. The numbers in horizontal axis corresponds to ligands in Table 1. Each row presents a protein binding site. There are (a) 1,165 and (b) 10,886 rows. The dendrograms in both axes were constructed by hierarchical clustering.

All docking scores of ligand-biding site pairs were merged into a matrix form. In total, yeast profiles and human profiles are composed of 1,165 and 10,886 binding sites, respectively with thirty-five ligands (Table 1). The numeric data are available in Additional files 1, 2. The resulting docking profiles were hierarchically clustered in both directions for the further analysis. The clustering results are shown in Figure 1.

**Table 1:** The list of ligands used to generate reverse docking profiles

Index	Ligand	PubChem CID
1	5-FC	3366
2	5-FU	3385
3	Brefeldin A	5287620
4	Camptothecin	24360
5	Chlorpromazine	2726
6	Cimetidine	2756
7	Clotrimazole	2812
8	Cycloheximide	6197
9	Dipyridamole	3108
10	Doxorubicin	31703
11	Dyclonine	3180
12	Fluvastatin	446155
13	Gemfibrozil	3463
14	Haloperidol	3559
15	Hydrocortisone	5754
16	Indomethacin	3715
17	Methotrexate	126941
18	Minoxidil	4201
19	Mitomycin C	5746
20	Morphine	5288826
21	Nifedipine	4485
22	Nitrofurantoin	6604200
23	Omeprazole	4594
24	Phenylbutazone	4781
25	Pravastatin	54687
26	Procaine	1548986
27	Progesterone	5994
28	Radicicol	6323491
29	Sulfamethoxazole	5329
30	Sulfinpyrazone	5342
31	Tamoxifen	2733526
32	Terbinafine	1549008
33	Theophylline	2153
34	Tunicamycin	6433557
35	Valproic acid	3121

The dendrograms along the column (chemical space) in both species are very similar to each other. We compared these dendrograms with two dendrograms from PubChem Structure Clustering [12] based on a measure recently developed for comparing two hierarchical clustering [13]. PubChem provides two kinds of clustering based on 2D structure fingerprint and 3D shape/feature similarity. Our two dendrograms are more similar with the clustering based on 3D similarity than that based on 2D similarity (Table 2). The reason may be that 3D conformations of ligands are more relevant for the protein-ligand docking fitness than 2D information. We also suggest that the topology of ligand clusters can be used as a new similarity measure between two small molecules. Nevertheless, relatively high similarity scores indicate that our docking profile data convey accurate information on their chemical properties.

**Table 2:** The similarities among hierarchical clusterings in ligand space.

	Human	Yeast	PC 2D	PC 3D
Human	1	0.939	0.513	0.708
Yeast	0.939	1	0.504	0.712
PC 2D	0.513	0.504	1	0.565
PC 3D	0.708	0.712	0.565	1

Rows and columns "Human" and "Yeast" represent clustering based on docking profile similarity in human and yeast, respectively. "PC 2D" and "PC 3D" represents clustering from PubChem based on 2D structure fingerprint and 3D conformation similarity.

### Druggablity analysis

The "druggability" of a certain target protein represents how probable the protein is in fact a real target of drugs, and it has been investigated in many previous studies [14-16]. In one such method, the druggability of a protein was inferred from its homologous proteins whose druggabilities were already known [17]. The weakness of this method is that the number of targets with known druggability is limited. Other approaches attempted to define "druggable" as "highly likely to bind to putative drugs", i.e., "bindability" [18,19].

**Figure 2:**
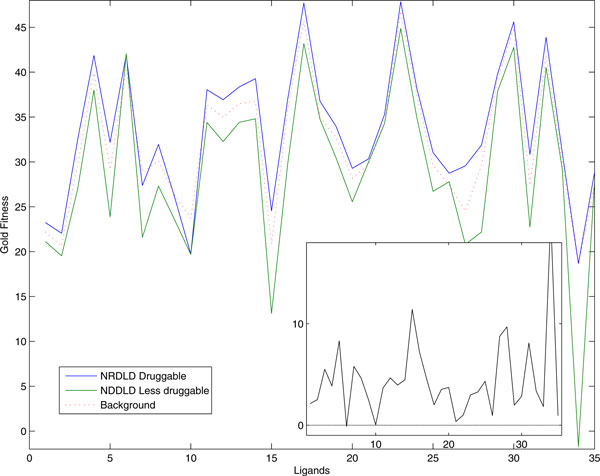
**Average docking scores of NRDLD druggable and less druggable set and those of overall docking fitness**. The numbers in horizontal axis represents the corresponding ligands in Table 1. The subplot represents difference between NRDLD druggable and less druggable (druggable minus less druggable). The values in subplot always show positive values except for small negative values (-0.13) in Ligand 6 (cimetidine).

In the context of bindability, the docking profiles in this study can provide good large scale simulated data. We first checked whether our data were in accord with predefined druggability dataset [20]. The non-redundant set of druggable and less druggable (NRDLD) set contains 71 druggable and 44 less-druggable targets. Since not all the entries are human proteins, the numbers of overlapped targets are 43 druggable and 8 less-druggable. Figure 2 shows the average docking fitness values of the sets of those overlapped targets, along with the averages of 10,886 binding sites' docking scores in human data set. Except for the case of cimetidine (Ligand 6), all averages of our docking scores of druggable set are greater than those of less-druggable set. One can observe that overall average values are placed between druggable and less-druggable sets in nearly all cases. This result suggests that without serious training it may be possible to classify all protein targets whose docking profiles are available into druggable or less-druggable target.

**Figure 3:**
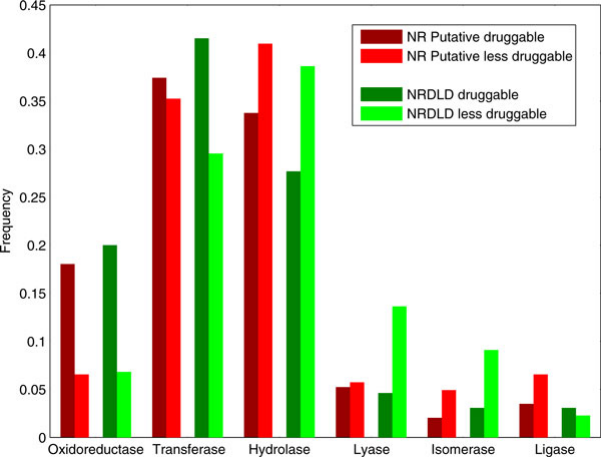
**Distributions of the enzyme classes**. Distributions of the enzyme classes in the NR Putative druggable and less druggable set (assigned in this study; red bars), and NRDLD druggable and less druggable set (green bars). Note that the enzyme class distribution trends are similar in both sets.

Next, by simply comparing docking fitness scores of each protein target against 35 ligands with the average fitness scores of those ligands (see Methods), we predicted 539 putative druggable binding sites and 289 less-druggable binding sites (Additional file 3). The predicted druggable and less-druggable binding sites were classified into 6 enzyme classes according to the first digit of their EC numbers [21] (Figure 3). Oxidoreductases occur more frequently in the druggable. Hydrolase, lyases and isomerases occur more frequently in the less-druggable set. Except for the case of ligase in which all relative frequencies are less than 0.1, the enzyme class distribution trends are similar in both the predicted set and NRDLD set.

### Protein function prediction based on docking profile similarity

**Figure 4:**
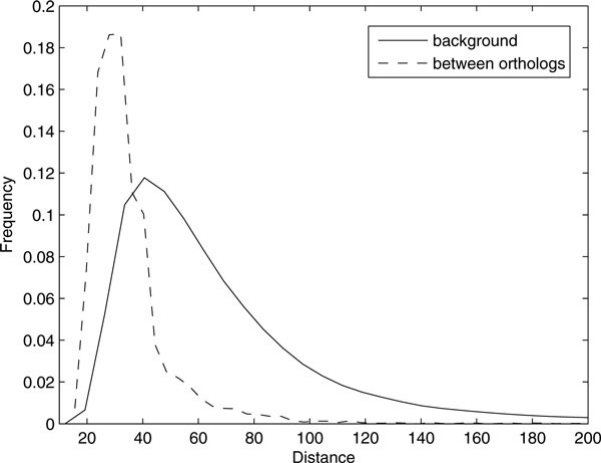
**Frequency distributions of distances**. The dashed line shows distribution of Euclidean distances of docking fitness between orthologs; the solid line shows that of distances between all yeast-human pairs.

An advantage of the present study is that docking profiles are available for both human and yeast proteomes. Yeast is the best characterized eukaryotic model organism. A variety of related resources such as chemical genomic profile [22], whole genome knock-out library [23], protein-protein interaction and genetic interaction data [24] are available. We expect that docking scores generated in this study can be combined with these resources to infer novel protein targets. In addition, it is valuable to check whether orthologous protein pairs of human and yeast share similar docking profiles. In Figure 4, we plot two distributions of Euclidean distances of docking scores of both species; one for the orthologous pairs and the other for all human-yeast pairs. It is observed that sequence similarity is generally reflected in the similarity of docking profile across the two species.

**Figure 5:**
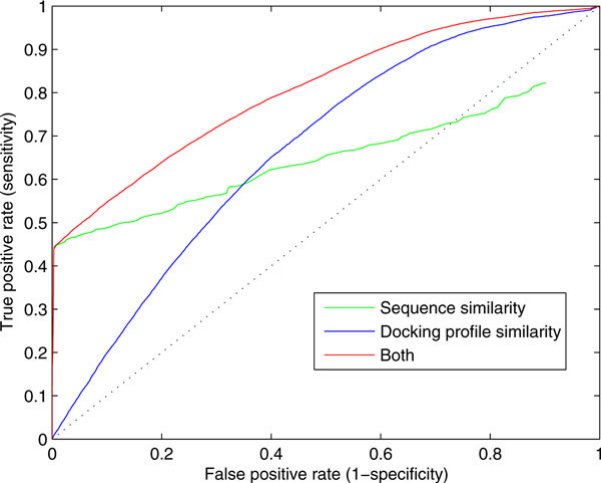
**ROC curve for assigning EC numbers (up to fourth digit) of proteins using those of nearest proteins in the other species**. Distances are based on BLAST e-value (sequence similarity), Euclidean distance of docking profiles (docking profile similarity), and hybrid of both. False positive rate is FP/(FP+TN), and true positive rate is TP/(TP+FN) where FP, TN, TP, FN are the numbers of false positives, true negatives, true positives, and false negatives, respectively.

The results shown in Figure 4 suggest that we can utilize our docking method to infer the function of proteins, especially the proteins that have no apparent orthologs with known function. To show that docking profiles contain the information which can be utilized to predict the function of proteins, we carried out a large-scale function prediction of enzymes. For 3,874,883 pairs of 5,989 human proteins and 647 yeast proteins, we collected all pairs for which EC numbers were available. We used BLAST e-value [25] as the sequence similarity measure, and Euclidean distance between the two docking profiles as the docking profile similarity measure. The performances are shown in Figure 5 as receiver operating characteristic (ROC) curve. In low false positive rate (FDR) region, using e-value yielded better performance, while docking profile similarity scores performed better in high FDR region. This limitation is due to substantial overlap between distance distributions of positive and negative pairs as shown in Figure 4. Although the average and median of distances of positive pairs are quite less than those of negative pairs, any single non-parametric Euclidean distance value cannot divide two groups perfectly. Relationship between docking fitness distance and enzyme function is more complex than a single threshold. However, it is observed that docking profile similarity contain positive information for function prediction which is not overlapped with the sequence information. Thus, this information would be used as a useful feature with combination of other features such as sequence similarity, structural similarity, and binding site similarity.

Here, for example, simple implementation of combination of sequence and docking profile information was tested. To cover low sensitivity of docking fitness in low FDR, a new distance was defined as follows: if BLAST e-value of a pair is less than 1e-5, e-value is used as the distance; if otherwise, Euclidean distance is used. The performance of this metric is shown in Figure 5 (red). Note that this simple metric is never based on any serious training, feature extraction, or machine learning technique. Not considering which elements in 35-dimentional docking profile are important, and simply adding information of docking profile exhibits better performances in all area. In summary, this implies that using docking profile information together with other useful measures as features of state-of-the-art machine learning technique and increasing the size of docking profile, i.e., appending the reverse docking results of additional ligands would get close to more precise function prediction of proteins.

### Case studies

The docking profile data generated in this study can be applied in a variety of ways. As discussed in the previous section, it can be utilized to infer protein function. On the other hand, more common application that has been explored in several previous studies is to infer new binding targets for known drugs. Here, we present two case studies.

### Binding target of 5-FC and 5-FU

5-fluorocytosine (5-FC) and 5-flurouracil (5-FU) are both fluorinated analogues of pyrimidine [26]. The structures of the two ligands are quite similar. Therefore, not surprisingly, the docking profiles are quite similar as well. Moreover, the top-ranking binding site of both ligands is the structure of yeast exosome component, the protein product of gene rrp6 (PDB id: 2hbm) [27]. The structure was identified relatively recently, so 2hbm has never been annotated as putative target, not to mention druggable. Previously known mechanism of action of 5-FU is inhibition of thymidylate synthetase [28]. Thus, the top-ranking structure, 2hbm, might be considered as a false positive. Fortunately, however, genome-wide study using tagged heterozygotes yeast mutants provided a strong evidence that rrp6 related rRNA processing exosome is a target of 5-FU [29]. The direct binding target of 5-FU was not identified in the previous study, but the result of that research and the docking scores strongly suggest that the protein product of rrp6 is the direct binding target of 5-FU in yeast.

### Protein structures from the same sequence

**Figure 6:**
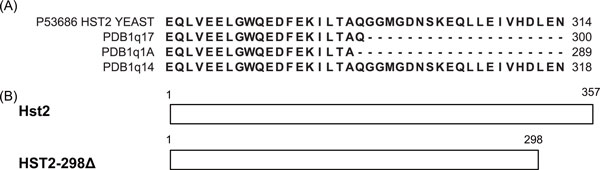
**Sequence variation among derivatives of yeast gene Hst2**. (a) C-termial sequences of intact Hst2 (uniprot ID: P53686) and those of its three structures. 1q17 and 1q1a are the structure of the 64 residue C-terminal deletion construct. (b) HST2-298Δ mutant strain only contains amino acids 1-298, with the deletion of C-terminal end. Regenerated from the previous work by others [33].

Similarly to the case of 5-FU, we also investigated the high-ranking targets in docking profile of cycloheximide (CHX). The top-ranking structure is the PDB structure 1q17 which is the protein structure of yeast gene Hst2, homologous to eukaryotic SIR2 [30]. Interestingly, among three protein products (1q17, 1q14, 1q1a) of hst2 whose structures were identified by the same researchers [31,32], 1q17 and 1q1a exhibited high binding fitness (1st and 6th ranked) while 1q14 showed poor binding affinity. We tried to find what caused these differences. It is known that 1q17 and 1q1a lack the 64 residue C-terminal tails of hst2 sequence in common while 1q14 is the structure of intact Hst2 (Figure 6a). We also found that there was the comparison study between yeast mutant strains, which lack corresponding C-terminal tail regions and wild type treating CHX [33] (Figure 6b). In that study, expression of HST2-298Δ which corresponds to PDB 1q17 or 1q1a led to increased sensitivity to CHX. This phenotype is surprisingly well characterized in docking profile in our study as the top-ranked docking fitness value.

### Are such a large number of protein structures necessary for reverse docking?

Compared to the protein sets used in previous studies, the set used in this study is quite large and has some redundancy. One may question whether all these structures contribute to the sensitivity of reverse docking. It is an important issue because docking still costs high computing power and is time-consuming.

In our dataset of human, 8,717 structures out of 10,886 structures have the hits sharing the same UniProt ID with 1,339 unique UniProt IDs. In other words, those 8,717 structures could be reduced into 1,339 structures by removing at most 7,378 structures if we filter the set with respect to only sequence redundancy. However, there are many cases where docking fitness profiles for similar sequences are quite different.

To show this property, we first carried out hierarchical clustering of docking profile of proteins. For each sub-cluster, if all the members were derivatives of the same UniProt ID, the members were merged into one. This procedure was repeated until there were no sub-clusters in which members shared the same UniProt ID. As a result, 1,710 structures were filtered out eventually, i.e., only about 20% of sequence-redundant protein structures exhibited the redundancy in docking profile. This is due to heterogeneity in PDB. There are many modified structures such as oxidized, reduced, multimeric, metal containing, and truncated forms for even a one protein sequence. Thus, we concluded that the sets of protein structures which were used in previous reverse docking studies are insufficient. For example, the interesting results from the docking of cycloheximide, which was discussed in the previous section, would have not been obtained.

Another interesting example is the main binding target of hydrocortisone, the glucocorticoid receptor (GCR). There are nine structures of the GCR in PDB. However, datasets used for reverse docking such as potential drug target database (PDTD) [6] included only two of them (PDB 1nhz and 1p93). The result of reverse docking of hydrocortisone by others [7] using PDTD could not detect the GCR as the target. In our docking profile, PDB 3bqd was the top-ranking protein target, which is another structure of the GCR. If we had removed redundancy based on sequence similarity, we could have not detected the real target of the GCR. Therefore, our reverse docking experiment suggests that using as many as possible protein structures in reverse docking is worthwhile in finding unknown drug targets or unexpected mode-of-action even though it costs high computation cost.

## Conclusions

In this study, we generated large-scale reverse docking profiles for all X-ray protein structures in human and yeast. These data can be the reference for future binding assays and used to find unexpected binding targets of drugs. Furthermore, it would be useful to find unknown therapeutic uses in drug repositioning. In some case studies, targets not annotated as druggable or not stored in target database previously exhibit high binding fitness and they are highly likely to be real binding targets considering previous functional experiments. By using a large number of protein structures, we improved the sensitivity of reverse docking and showed that using as many protein structure as possible was important in finding real binding targets. Although we used as small as 35 ligands in docking, we were able to demonstrate some usefulness of our data. Generating this kind of reverse docking profile of a large number of ligands would be valuable in the future study.

## Methods

### Data preparation

All available X-ray protein structures in human and budding yeast *Saccharomyces cerevisiae *were retrieved from RCSB Protein Data Bank (PDB) [34,35]. The best putative binding sites of each PDB structure were generated by using the program Fpocket [36,37]. To make pockets appropriate inputs for the docking, Open Babel [38] was used to protonate all the pockets. Thirty-five well-known ligands (Table 1) were manually selected from previous high-throughput experimental studies [29,39] to perform high-throughput reverse docking after excluding some ligands that were too large or small for molecular docking study. The 3D structures of the ligands were retrieved from PubChem [40] and converted from sdf file [41] into Tripos mol2 file format.

### Docking

All the protonated pockets were docked against the ligand set using GOLD [42]. We used a 'flexible ligand-rigid protein' mode. All other options involved in GOLD's search algorithm and termination factor were set to the default options. Given several putative docking conformations, we only chose the highest-ranking binding pose for each ligand-biding site pair. The GOLD fitness value [43] was used as a measure of the binding fitness. As a result, 10,886 × 35 matrix and 1,165 × 35 matrix of docking fitness scores for human and yeast, respectively, were made and used in this study (Additional files 1, 2).

### Druggability analysis

Predefined the non-redundant set of druggable and less druggable binding sites (NRDLD set) was retrieved from the study by Krasowski et al. [20]. Among 71 druggable binding sites and 44 less-druggable ones in NRDLD set, 43 druggable and 8 less-druggable binding sites are overlapped with human protein structures used in this study. These 51 binding sites were used for druggability analysis.

Putative druggable and less-druggable protein binding sites were assigned by the following rules: a binding site is druggable when all 35 docking values of the binding site are always larger than corresponding overall average values, and less-druggable when all 35 docking values of a binding site are always less than corresponding average values.

To get EC number composition of assigned druggable and less-druggable sets, non-redundant (NR) putative druggable and less druggable sets were defined. In this study, NR set means that the set do not contain any pairs of proteins sharing the same UniProt ID [44]. Note that we did not use any sequence identity measure to remove redundancy.

### Ortholog mapping

Although there are several ortholog databases, none of those provides PDB-based mapping table. Therefore, we obtained the ortholog mapping between human and yeast protein structures by the following procedure. First, we retrieved human-yeast ortholog table from InParanoid [45,46]. In this table, human proteins and yeast proteins were annotated by Ensembl's id (ENSP) [47] and yeast ORF name [48], respectively. These terms were transferred into PDB id by PICR [49] to complete PDB-based mapping.

## Competing interests

The authors declare that they have no competing interests.

## Authors' contributions

ML carried out docking, ortholog analysis, druggability analysis and case studies. DK designed this work and contributed to draft the manuscript. All authors read and approved the final manuscript.

## Supplementary Material

Additional file 1 **Docking profiles for human protein structures**. A csv document containing all docking scores of human protein structures with ligands used in this study. This file can be viewed with Microsoft Excel or any text editor. Click here for file

Additional file 2 **Docking profiles for yeast protein structures**. A csv document containing all docking scores of yeast protein structures with ligands used in this study. This file can be viewed with Microsoft Excel or any text editor. Click here for file

Additional file 3 **The list of putative drugggable and less druggable targets based on docking profile**. The list of putative drugggable and less druggable targets based on docking profile. The file contains PDB ID, corresponding EC number, and assigned druggability. This file can be viewed with Microsoft Excel or any text editor. Click here for file
